# Genomic signatures of population bottleneck and recovery in Northwest Atlantic pinnipeds

**DOI:** 10.1002/ece3.4143

**Published:** 2018-06-05

**Authors:** Kristina M. Cammen, Thomas F. Schultz, W. Don Bowen, Michael O. Hammill, Wendy B. Puryear, Jonathan Runstadler, Frederick W. Wenzel, Stephanie A. Wood, Michael Kinnison

**Affiliations:** ^1^ School of Marine Sciences University of Maine Orono ME USA; ^2^ Duke University Marine Lab Nicholas School of the Environment Beaufort NC USA; ^3^ Bedford Institute of Oceanography Dartmouth NS Canada; ^4^ Fisheries and Oceans Canada Maurice Lamontagne Institute Mont‐Joli QC Canada; ^5^ Department of Infectious Disease and Global Health Cummings School of Veterinary Medicine Tufts University North Grafton MA USA; ^6^ Protected Species Branch, NOAA, NMFS Northeast Fisheries Science Center Woods Hole MA USA; ^7^ Department of Biology University of Massachusetts Boston MA USA; ^8^ School of Biology and Ecology University of Maine Orono ME USA

**Keywords:** approximate Bayesian computation, gray seal, harbor seal, restriction site‐associated DNA sequencing

## Abstract

Population increases over the past several decades provide natural settings in which to study the evolutionary processes that occur during bottleneck, growth, and spatial expansion. We used parallel natural experiments of historical decline and subsequent recovery in two sympatric pinniped species in the Northwest Atlantic, the gray seal (*Halichoerus grypus atlantica*) and harbor seal (*Phoca vitulina vitulina*), to study the impact of recent demographic change in genomic diversity. Using restriction site‐associated DNA sequencing, we assessed genomic diversity at over 8,700 polymorphic gray seal loci and 3,700 polymorphic harbor seal loci in samples from multiple cohorts collected throughout recovery over the past half‐century. Despite significant differences in the degree of genetic diversity assessed in the two species, we found signatures of historical bottlenecks in the contemporary genomes of both gray and harbor seals. We evaluated temporal trends in diversity across cohorts, as well as compared samples from sites at both the center and edge of a recent gray seal range expansion, but found no significant change in genomewide diversity following recovery. We did, however, find that the variance and degree of allele frequency change measured over the past several decades were significantly different from neutral expectations of drift under population growth. These two cases of well‐described demographic history provide opportunities for critical evaluation of current approaches to simulating and understanding the genetic effects of historical demographic change in natural populations.

## 1 INTRODUCTION

Many species today have been broadly shaped by a shared history of population decline followed, in some cases, by subsequent recovery. Historically, large changes in the abundance and distribution of wildlife populations have resulted from both natural (e.g., large‐scale climatic shifts, Boehme et al., 2012) and anthropogenic (e.g., exploitation followed by protection, Lotze, Coll, Magera, Ward‐Paige, & Airoldi, 2011) factors. These demographic changes can have long‐lasting effects beyond the number and location of individuals and are particularly important in shaping extant genetic diversity (Wright, 1931). Reductions in population size, and particularly events that lead to geographic isolation and fragmented distributions, can drastically reduce genetic diversity (Nei, Maruyama, & Chakraborty, 1975).

On the other side of this demographic trajectory, evolutionary biologists have theorized that growing populations should experience a rise in genetic diversity over time, at a base level of the rate of mutation in closed populations (Nei et al., 1975) and at a higher rate with contributions from migrants in an open population or metapopulation system (Hansson et al., 2000; Keller et al., 2001). Furthermore, geographically expanding populations are predicted to vary spatially, with lower overall genetic diversity at the edge of their expansion wave and differences in the frequency of specific genetic variants as a result of sampling bias or selection for certain traits in successful migrants (reviewed in Excoffier, Foll, & Petit, 2009).

These evolutionary processes that occur during bottleneck, growth, and spatial expansion have been primarily explored through simulated datasets that undergo artificial changes in population size and number. From such simulations, we have learned how factors such as mating systems (Armburster & Pfenninger, 2003), epistasis (Turelli & Barton, 2006), baseline allele frequency distributions (Luikart, Allendorf, Cornuet, & Sherwin, 1998), intensity and length of bottleneck (England et al., 2003), and timing of recovery (Hoban, Gaggiotti, & Bertorelle, 2013) are likely to affect the loss of genetic diversity during bottlenecks. More recent studies that compare the power of traditional and genomic markers to detect bottlenecks also show us that the type and amount of data and the selected model parameters can significantly impact conclusions (Cabrera & Palsbøll, 2017; Elleouet & Aitken, 2018; Hoban et al., 2013; Peery et al., 2012; Shafer, Gattepaille, Stewart, & Wolf, 2015). While advances in computational power have enabled increasingly complex demographic models and the incorporation of Bayesian approaches provide more nuanced ways to draw predictions and interpret uncertainty, simulated datasets inherently lack natural variability that without a doubt influences these processes in the natural environment. This gap cannot be fully addressed by laboratory experiments in model systems, which have been used to test some simulated expectations (e.g., England et al., 2003; Spencer, Neigel, & Leberg, 2000). Rather, to address this gap, we identify wildlife populations with well‐described histories of bottleneck and recovery (i.e., conservation success stories) as ideal systems in which to study these evolutionary processes embedded in natural variability. In these populations, we test hypotheses informed by theoretical expectations that historical reductions in population size will leave signatures of genomic bottleneck, cohorts sampled throughout recovery will show increasing levels of genomic diversity, and genomic diversity will also change along axes of range expansion.

**Figure 1:**
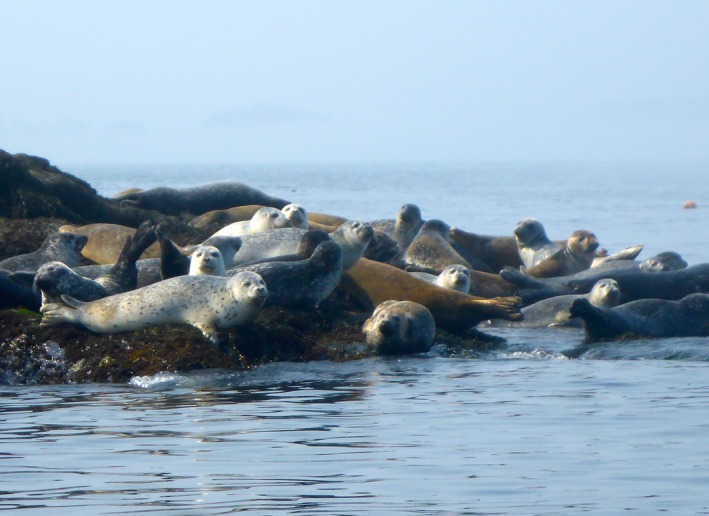
Harbor seals (*Phoca vitulina vitulina*) at a haulout site in Maine

Sympatric pinniped species in the Northwest Atlantic, the gray seal (*Halichoerus grypus atlantica*) and harbor seal (*Phoca vitulina vitulina*, Figure 1), provide parallel natural experiments of historical decline and subsequent recovery through which we can investigate the impact of recent demographic change in genomic diversity. These sympatric populations share relatively similar demographic histories that include large changes in abundance and distribution coincident with glacial retreat and recent anthropogenic impacts. This demographic history is described in climatic, archeological, and historical records. Low availability of continental shelf habitat in the North Atlantic during the last glacial maxima suggests gray and harbor seal populations may have been small (~15,000–21,000 gray seals) until approximately 12,000 years ago when habitats expanded following glacial retreat and the populations accordingly increased (Boehme et al., 2012). Historical records thereafter suggest an abundance of seals in the Northwest Atlantic in the 16th century when European explorers arrived (reviewed in Lavigueur & Hammill, 1993), but by the mid‐20th century, subsistence hunting, government‐sponsored bounties (Bowen & Lidgard, 2013; Lelli, Harris, & Aboueissa, 2009), and commercial exploitation (Mowat, 1984) had drastically reduced both seal populations. At that point, gray seals were considered rare in both eastern Canada and the Northeast United States (Davies, 1957), and harbor seal pupping colonies had been extirpated south of Maine (Katona, Rough, & Richardson, 1993). Following the cessation of bounties, enactment of local protection (Lelli & Harris, 2006), and passing of the US Marine Mammal Protection Act of 1972, population surveys have documented the rapid return of these seals over the past several decades (Bowen, den Heyer, McMillan, & Hammill, 2011; Gilbert, Waring, Wynne, & Guldager, 2005; Waring, Josephson, Maze‐Foley, & Rosel, 2016). In the Northeast United States, estimates of harbor seal population size have grown from 5,000 in the early 1970s (Richardson, 1976) to over 75,000 today (Waring et al., 2016), and gray seals have returned from essentially absent until the early 1990s (Gilbert et al., 2005) to 30,000–50,000 today (Moxley et al., 2017).

The recovery trajectories of these seals have differed within and between taxa, providing fodder for evolutionary analyses that investigate the varying effects of reproduction and migration as well as the shift from exponential growth toward carrying capacity (Gaggiotti et al., 2002). Today, growth at the largest worldwide gray seal population on Sable Island in Canada is slowing potentially as a result of nearing carrying capacity (den Heyer, Lang, Bowen, & Hammill, 2017), while gray seal populations in the United States continue to increase (Waring et al., 2016), likely representing a population size‐mediated range expansion from Canadian source populations (Wood et al., 2011). Following protection, the harbor seal population in the Northeast United States also experienced steady growth through the 1990s (Gilbert et al., 2005), but more recently this growth has slowed and potentially reversed (Waring et al., 2016). Even more dramatic, on Sable Island the rapid growth of gray seals has been coincident with a reduction to near absence of the local harbor seal population (Bowen, Ellis, Iverson, & Boness, 2003; Bowen, McMillan, & Mohn, 2003).

These historical and recent trends mirror those in the Northeast Atlantic, where historical exploitation led to local extirpation of seal colonies, but the past several decades have been generally characterized by recovery. Today, most gray seal colonies are stable or increasing, although recent declines have been recorded in sympatric harbor seal populations (Hall & Kershaw, 2012). Prior analyses that compare genetic diversity of archeological and contemporary samples in this region reveal a shift in subspecies boundary following extirpation of gray seal colonies around Denmark and mixed recolonization from both Baltic and Eastern Atlantic subspecies (Fietz et al., 2016). On a more recent temporal scale, genetic analyses have also been used to monitor gray seal recolonization of islands in the Orkney archipelago north of Scotland, where there exists fine‐scale genetic population structure on the order of tens of kilometers (Gaggiotti et al., 2002). Beyond the observed impacts of recolonization on subspecies and population structure, these studies generally report high levels of genetic diversity, suggesting little impact of historical bottleneck. In fact, recent analyses find stronger evidence for signatures of postglacial expansion than recent bottlenecks in mitochondrial and microsatellite data from gray seals in the Baltic and Northeast Atlantic (Klimova et al., 2014).

In comparison with the relatively rich history of regional genetic studies of gray seals (Allen, Amos, Pomeroy, & Twiss, 1995; Cammen, Hoffman, Knapp, Harwood, & Amos, 2011; Gaggiotti et al., 2002) and to a slightly lesser extent harbor seals (Goodman, 1998; Olsen et al., 2017) in the Northeast Atlantic, there exists relatively little parallel analysis of gray or harbor seals in the Northwest Atlantic, and few attempts to model or monitor long‐ or short‐term histories using genetic tools. Samples from this region have been occasionally incorporated into worldwide or ocean basin analyses (Cammen et al., 2011; Klimova et al., 2014; Stanley et al., 1996), but within the Northwest Atlantic, most seal genetics studies have focused on a single colony (Coltman, Bowen, & Wright, 1998; Worthington Wilmer, Allen, Pomeroy, Twiss, & Amos, 1999). One of only two published regional analyses of gray seals in the Northwest Atlantic used mitochondrial and microsatellite markers to study population structure and recolonization and reported panmixia and migration from Canadian source populations to the growing colonies in the Northeast United States (Wood et al., 2011). A recent comparison of archeological and contemporary samples of both gray and harbor seals in the Northwest Atlantic quantified the loss of genetic diversity in the mitochondrial control region of both species over the past 500 years resulting from human exploitation (Cammen et al., 2017).

Expanding upon this recent study, here, we apply a high‐throughput genotyping‐by‐sequencing approach to biological collections from the past half‐century to test for genomic signatures of population bottleneck and recovery. In doing so, we attempt to address a current gap in pinniped genomic studies, many of which explore population structure, but few of which test for historical demographic patterns (Klimova et al., 2014). From very recent estimates of extant genomic diversity, we project backwards using approximate Bayesian computation (ABC) to describe lingering genomic signatures of historical changes in population size. From estimates of genomic diversity sampled early in the recovery process, we project forwards using a Wright–Fisher model of genetic drift in a population experiencing logistic growth to evaluate recovery of genomic diversity over the past half‐century. Together, these analyses provide a valuable assessment of theoretical and simulated changes in genetic diversity during population decline and growth in natural populations.

## 2 METHODS

### 2.1 Samples

We assessed a subset of the samples analyzed for mitochondrial control region variability in Cammen et al. (2017), including 252 gray seals and 55 harbor seals of good to excellent DNA quality (Table 1). The strategy of larger sample sizes, at the expense of lower read depth per sample, was selected based on the recommendation of prior simulation studies that suggest better performance for demographic estimation (Elleouet & Aitken, 2018) and inferring diversity and population structure (Fumagalli, 2013). The difference in total number of samples between gray and harbor seals reflects the geographic and temporal expanse of sampling and is driven by sample availability from prior long‐term studies; however, the number of samples per cohort was approximately equivalent across sites and species.

**Table 1:** Summary of genomic diversity assessed by RAD sequencing among gray and harbor seals from the Northwest Atlantic. Values are presented as the mean (and standard deviation) per locus per sample group for allelic richness corrected by rarefaction to the sample size of the smallest cohort or colony (A_R_) and expected heterozygosity (H_E_)

Species	Location	Cohort	*N*	A_R_	H_E_
Gray seal	Gulf of St Lawrence	2015	32	1.83 (0.38)	0.20 (0.17)
	Sable Island	All	153	1.84 (0.27)	0.20 (0.17)
		1973–74	28	1.81 (0.39)	0.21 (0.18)
		1985	29	1.82 (0.38)	0.21 (0.17)
		1998	32	1.83 (0.35)	0.21 (0.17)
		2004	32	1.82 (0.37)	0.21 (0.18)
		2015	32	1.82 (0.36)	0.21 (0.18)
	Muskeget Island	All	67	1.84 (0.32)	0.20 (0.17)
		2002	33	1.66 (0.47)	0.16 (0.18)
		2015–16	34	1.67 (0.46)	0.17 (0.18)
	Total		252		
Harbor seal	Northeast United States	1992–98	11	1.62 (0.49)	0.16 (0.18)
		2003–05	19	1.58 (0.42)	0.16 (0.18)
		2013–15	25	1.58 (0.40)	0.16 (0.18)
	Total		55		

**Figure 2:**
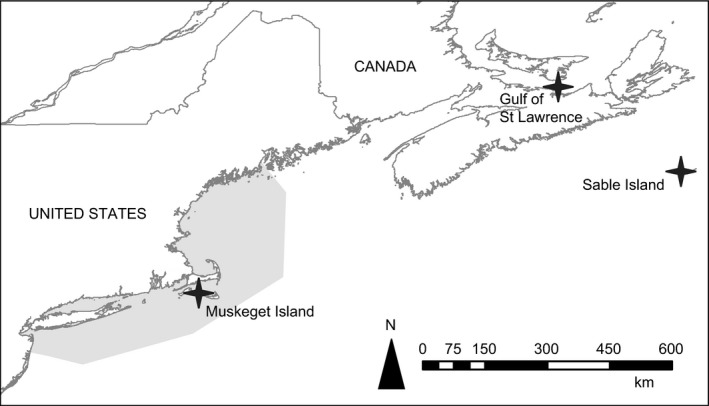
Map of seal sampling sites. Gray seals were sampled at three breeding colonies indicated by stars. By‐caught harbor seals were sampled throughout the shaded area off the Northeast United States

Gray seal samples were previously collected as part of long‐term studies at three breeding colonies in the Northwest Atlantic: Sable Island, the Gulf of St. Lawrence, and Muskeget Island (Figure 2). Only samples collected from either pups or known‐aged individuals were included so that individuals could be assigned to known cohorts that spanned the time of exponential population growth on both Sable (1973–74, 1985, 1998, 2003, 2015) and Muskeget (2002, 2015–16) islands. Sampling of live harbor seals in this region over this time period has been limited, so we relied on collections from seals that had been by‐caught in commercial fisheries in the Northeast United States between 1992 and 2015 (Figure 2). Only samples collected from either pups or yearlings were included so that individuals could be assigned to one of three cohorts. These samples cannot be assigned to their natal breeding colony, but based on location and knowledge of movement from prior tagging research (Waring, Gilbert, Loftin, & Cabana, 2006), these seals likely originated from breeding colonies along the coast of Maine; only a few harbor seals of known Canadian origin (based on attached flipper tags) have been observed by‐caught in the Northeast United States since 1990.

### 2.2 RAD sequencing

We conducted double‐digest restriction site‐associated DNA (ddRAD) sequencing following the protocol of Peterson, Weber, Kay, Fisher, and Hoekstra (2012) with minor modifications described in detail in the Appendix S1. In contrast to the original RAD protocol (Baird et al., 2008), ddRAD does not require manual shearing of the DNA and produces generally fewer loci per genome, which was preferable for laboratory and analytical efficiency with large sample sizes. Restriction enzymes SBfI‐HF and MspI were selected for DNA digestion to sequence approximately 100,000 loci per genome, estimated via an *in silico* digest of the closely related Weddell seal (*Leptonychotes weddellii)* genome, LepWed1.0 (Assembly GCF_000349705.1), using a wide size range (150–500 bp) to reflect size selection by 1.5× magnetic beads. In total, 10 RAD libraries were built and sequenced on five lanes of an Illumina HiSeq 2500 using 50‐bp single‐end read sequencing. Samples from each group were distributed approximately evenly across multiple libraries (32 individuals per library with unique P1 barcodes) and sequencing lanes (two libraries per lane with unique P2 index markers), to avoid any library or lane effects. RAD libraries were generated at the Duke University Marine Conservation Molecular Facility and sequenced by the Duke Center for Genomic and Computational Biology.

Resulting sequences were processed with Stacks v1.21 (Catchen, Amores, Hohenlohe, Cresko, & Postlethwait, 2011) to identify and genotype SNPs *de novo* (without a reference genome). Sequences were first filtered for quality (minimum average phred score of 10 across 8‐bp sliding window; no sequencing errors in the barcode or restriction enzyme site) and demultiplexed by P2 index and P1 barcode. The program then builds a catalog of RAD loci with user‐defined parameters through “stacking” sequence reads that putatively originated from the same location in the genome.

The selection of parameters can have significant effects on downstream analyses and subsequent conclusions (Paris, Stevens, & Catchen, 2017; Shafer et al., 2017). We therefore systematically selected Stacks parameters to optimize the number of polymorphic loci using the *r80* optimization approach described in Paris et al. (2017). Optimization tests were run on a subset of 30 individuals of each species, distributed equally such that the optimization datasets included 10 individuals from each harbor seal cohort and 10 individuals from each gray seal colony (and an equal number from each cohort within the colony, if relevant). All libraries and sequencing lanes were represented in this optimization dataset, and the samples represented the full range in sequence coverage per individual. We tested values of *m* (the minimum number of identical raw reads required to create a stack) from 1 to 6, *M* (the minimum number of mismatches allowed between loci when processing a single individual) from 0 to 6, and *r* (minimum percentage of individuals per population) of 0.4, 0.6, and 0.8, using default values of *m *=* *3, *M *=* *2, and *n *=* *0. We evaluated the effects of *m* and *M* parameter selection on the number of assembled loci, the number of polymorphic loci, and the number of SNPs for each value of *r*. We also evaluated the effects of *m* on stack depth, or coverage. An optimal value of *m* was chosen that produced the highest number of polymorphic loci and SNPs. As the number of SNPs continued to rise with increasing *M* in our datasets, we selected an optimal *M* after which point there was little increase in polymorphic loci, in order to balance trade‐offs between increasing detection of true polymorphisms and incorrectly identifying sequencing error in overmerged loci as *M* increases. Following Paris et al. (2017)'s recommendation, we set *n* (the minimum number of mismatches allowed between loci when building the catalogue) equal to *M*.

With the selected optimal parameters, we built two catalogs of RAD loci, one for each species. The harbor seal RAD locus catalog was built using sequence data from all 55 individuals, but for computational efficiency, it was necessary to build the gray seal catalog from a subset of individuals. The gray seal catalog was built using sequence data from 7 individuals from each sample group (cohort/population), distributed evenly across RAD libraries and sequencing runs and selecting for individuals with the greatest coverage, for a total of 56 individuals. Highly repetitive RAD loci, characterized as any loci with a stack depth greater than two standard deviations from the mean, as well as all stacks that differ from these repetitive loci by only one nucleotide, were removed to reduce the potential effects of PCR duplicates and fragment size bias. SNPs were detected within each stack in the catalogs using a bounded maximum‐likelihood model (Catchen, Hohenlohe, Bassham, Amores, & Cresko, 2013).

The consensus sequences of all RAD loci from both gray and harbor seals were independently compared to the Weddell seal genome to identify overlap in loci between gray and harbor seals. Alignments were carried out using Bowtie v0.12.9 (Langmead, Trapnell, Pop, & Salzberg, 2009), accepting only alignments with less than three mismatches and a single best match to the genome. There is currently no publicly available reference genome for either study species, but Weddell seals diverged from gray and harbor seals <24 million years ago (Arnason et al., 2006), and high rates of mapping success for both species confirm its suitability for our analysis.

### 2.3 Historical demography—bottleneck period

**Figure 3:**
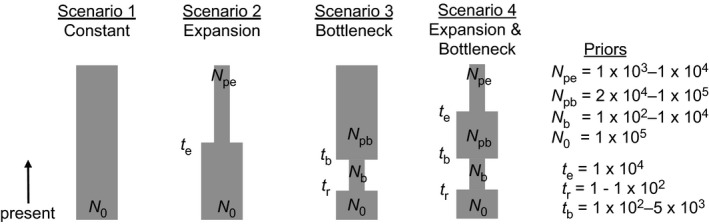
Demographic scenarios, with priors, tested with approximate Bayesian computation using DIYABC. Scenarios are defined by relative changes in effective population size (*N*) at given times (*t*) since present

We estimated historical changes in population size from a subset of the genetic data using ABC analyses implemented in DIYABC v2.1.0 (Cornuet et al., 2014) with one million simulated datasets representing four different demographic models (Figure 3): (1) constant population size over time (null hypothesis), (2) expansion, (3) bottleneck, and (4) expansion followed by bottleneck. We used scaled parameters, to avoid the potential losses in accuracy or increases in error that have been observed in prior simulations comparing scaled and unscaled parameters (Cabrera & Palsbøll, 2017). This approach, which reveals relative changes in population size at relative times from present, further avoids incorporating uncertainties in generation time and estimates of absolute population size into the models, and allows us to use the same parameters for both species, enabling a direct comparison. Estimates of effective population size were scaled relative to the present size *N*_0_ = 100,000, and times of demographic change were scaled relative to the oldest time point used in our models, the end of the last glacial maximum at *t*_*e*_ = 10,000.

The expansion event (in models 2 and 4) was parameterized to reflect hypothesized expansion in seal habitat in the North Atlantic following the end of the last glacial maximum. Given estimates of available, suitable habitat, Boehme et al. (2012) estimated that gray seal population sizes in the North Atlantic during the last ice age were 1 to 1.5 orders of magnitude smaller than today. Given similar habitat needs for gray and harbor seals, we used a uniform prior between 1,000 and 10,000 to estimate pre‐expansion population size (*N*_pe_) for both species. The bottleneck event (in models 3 and 4) was parameterized to reflect recent human exploitation and bounties. We used a uniform prior between 100 and 5,000 for the timing of the bottleneck (*t*_*b*_) to span exploitation by Native Americans and Europeans starting up to 9,000 years ago and peaking in the 17th and 18th centuries (Lavigueur & Hammill, 1993), as well as the government‐sponsored bounties that followed (Bowen & Lidgard, 2013; Lelli et al., 2009). We used a uniform prior between 1 and 100 for the timing of recent recovery (*t*_*r*_) following the end of the bounties and beginning of local and federal protections. The magnitude of the bottleneck across the Northwest Atlantic is uncertain, but in the Northeast United States, gray seals were largely absent in the mid‐1900s and one of the first harbor seal population surveys in the early 1970s estimated only 5,000 individuals (Richardson, 1976). Given the current estimates of ~300,000 gray seals and 70,000–100,000 harbor seals in the Northeast United States (Waring et al., 2016), we used a uniform prior between 100 and 10,000 for bottleneck size (*N*_*b*_). Finally, there remains significant uncertainty (and ongoing debate) regarding the size of the prebottleneck population size (*N*_pb_), so we used a broad uniform prior between 20,000 and 100,000, suggesting the species may have been approximately equally abundant as they are today, or up to five times less abundant prior to human impact and subsequent recovery.

We assessed the likelihood of these demographic models in each of the most recent gray and harbor cohorts independently, using 1,000 RAD loci sequenced in at least 90% of the individuals, to reduce potential impacts of missing data. Only one SNP per RAD locus was included in this analysis, to reduce biases previously observed in simulations with four segregating SNPs per locus as compared to a single SNP (Shafer et al., 2015). Models were tested both with and without a minimum minor allele frequency (MAF) filter of 0.05; rare minor alleles are often removed from downstream RAD analyses as a conservative measure to remove sequencing errors, but rare alleles can also be particularly informative for studies of demography (Keinan & Clark, 2012; Maruyama & Fuerst, 1985a,1985b).

We assessed the likelihood of each model using four summary statistics: proportion of zero values, mean and variance of nonzero values, and mean of complete distribution. The posterior probability of each demographic model was estimated using logistic regression on the top 1% of the simulated datasets based on similarity of summary statistics to the observed values. The error associated with model selection was calculated as the probability of selecting a given model when it is not true, that is, its incorrect selection among 1,000 datasets simulated for each of the alternate models (Cornuet, Ravigné, & Estoup, 2010). Finally, we estimated the posterior probabilities of the model parameters (effective population sizes and times of population size change) using a logit‐transformation and the top 1% closest simulated datasets.

### 2.4 Historical demography—recovery period

To assess recovery rates of genomewide diversity following population bottleneck over the past several decades, we evaluated the average allelic richness (A_R_) and expected heterozygosity (H_E_) for each cohort sampled within a colony. This analysis included SNPs sequenced in at least 80% of individuals in all cohorts within a colony and was carried out using diveRsity (Keenan, McGinnity, Cross, Crozier, & Prodohl, 2013) in R. A_R_ was calculated with rarefaction to standardize to the sample size of the smallest cohort. We also compared these metrics of genomewide diversity among colonies (with cohorts combined) to assess changes in diversity along the axis of spatial expansion during this period of recovery. We further evaluated extant population structure in both species following expansion using fastSTRUCTURE (Raj, Stephens, & Pritchard, 2014), which implements a Bayesian approach to testing user‐specified values of *K* (1–3), the number of significantly supported clusters, with no *a priori* information about sample origin. Spatial and temporal analyses were then merged in a hierarchical analysis of molecular variance (AMOVA) implemented in Arlequin v3.5 (Excoffier & Lischer, 2010).

We further compared observed rates of change in allele frequency to those predicted under neutral expectations of genetic drift in a single population experiencing logistic growth. Neutral expectations were generated using a forward simulator built for high‐throughput sequencing genomic datasets, popRangeSim (McManus, 2015) in R. We made minor modifications to the source code to model logistic growth as *N*_*t*+1_ = *N*_*t*_ + *rN*_*t*_(1−*N*_*t*_/*K*), where *N* is the population size at present (*t*) and following one generation of growth (*t*+1), *r* is the population growth rate, and *K* is the carrying capacity. popRangeSim implements a Wright–Fisher approach using the binomial distribution of probabilities to model stochastic genetic drift each generation prior to population growth. We did not incorporate mutation, given its likely minimal impact over the time period assessed here.

For Sable Island gray seals, for which we have the longest dataset of population surveys and genetic samples, the model was parameterized using the following values informed by the literature to represent the time period 1970 to 2015: starting population size = 5,000 (Bowen, McMillan, & Mohn, 2003); *r *=* *.13 for 25 years followed by *r *=* *.04 for the subsequent 20 years (den Heyer et al., 2017); and *K *=* *500,000 (Hammill, den Heyer, Bowen, & Lang, 2017). The model was initially seeded with observed SNP frequencies at all *r80* loci from the 1973 to 1974 cohort and run for 45 years, sampling allele frequencies at time points that corresponded to the sequenced cohorts. Similarly, to assess rates of change in allele frequency among harbor seal cohorts, the model was parameterized as follows to represent the time period 1995 to 2015: starting population size = 67,500; *r *=* *.066 (Gilbert et al., 2005); and *K *=* *100,000 (Waring et al., 2016). The model was initially seeded with SNP frequencies at all *r80* loci from the 1992 to 1998 cohort and run for 20 years, sampling allele frequencies every 10 years. We calculated the degree (slope) and strength (*p*‐value) of temporal linear regressions for all empirical and simulated loci and considered loci with slopes that exceeded the maximum slope of all simulated loci with a corresponding *p*‐value <.05 to be significant outliers.

## 3 RESULTS

**Figure 4:**
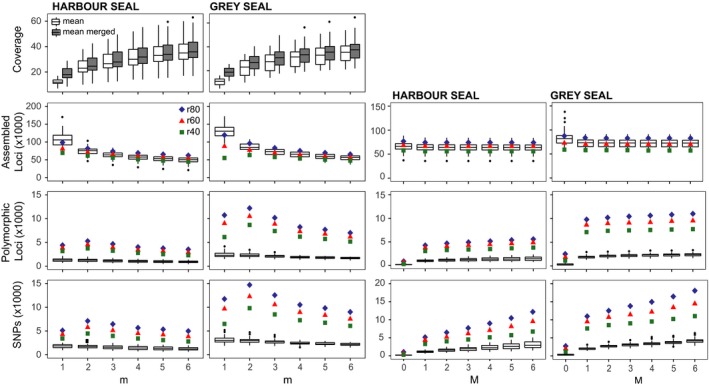
Evaluating the effects of varying Stacks parameters, *m*,* M*, and *r*, on average read coverage and number of assembled loci, polymorphic loci, and SNPs. Values up to 6 were tested for *m*, the minimum number of identical raw reads required to create a stack, and *M,* the minimum number of mismatches allowed between loci when processing a single individual. Values of 80% (blue diamonds), 60% (red triangles), and 40% (green squares) were tested for *r,* the minimum percentage of individuals per population. Boxplots depict the median values (and quartiles) for 30 individuals of each species

RAD sequencing resulted in a total of 628.4 million gray seal reads and 111.5 million harbor seal reads, with an average of 2.4 (±1.4 *SD*) million reads per individual, following the implementation of sequence quality filters. The systematic evaluation of Stacks parameters, following Paris et al. (2017), identified similar optimal parameters for both species. As expected, and previously demonstrated in Paris et al. (2017), the mean coverage per locus increased and the number of assembled loci per individual decreased as *m* increased for both species (Figure 4). The number of polymorphic loci and SNPs for both gray and harbor seals was greatest at *m *=* *2, which was therefore selected as the optimal value for both datasets. Changing the *M* parameter had little impact on the number of assembled loci, although we observed a slight drop after *M *=* *1 (Figure 4). The number of polymorphic loci and SNPs jumped from *M *=* *1 to 2 and then continued to rise for greater values of *M*. After the transition from *M *=* *1 to 2, the number of polymorphic loci rose by fewer than 150 loci with each incremental increase of *M*, and we therefore selected *M *=* *2 and *n = M = *2 as the optimal value for both datasets.

Interestingly, it was clearly apparent in our evaluation of Stacks parameters that the gray seal data set was significantly more polymorphic than the harbor seal dataset. Gray seals had more than twice the number of polymorphic loci and SNPs than harbor seals at the selected *m *=* *2 for any tested value of *r*, and this trend remained across all tested values of *m* and *r* (Figure 4). Similarly, across all tested values of *M* and *r*, gray seals had twice, or nearly twice, the number of polymorphic loci and a greater number of SNPs.

Using the selected, optimal parameters and following the removal of highly repetitive stacks, gray and harbor seal RAD catalogs contained 244,822 and 195,906 *de novo* loci, respectively. RAD loci of the two species were compared through alignment to the reference Weddell seal genome. For gray seals and harbor seals, respectively, 187,696 (76.67%) and 145,591 (74.32%) loci aligned to the Weddell seal genome, and 100,251 loci were sequenced in both study species.

We identified, genotyped, and further analyzed 8,716 and 3,761 polymorphic *r80* loci containing 10,097 and 4,312 SNPs that were sequenced in at least 80% of the individuals in at least one sample group in gray and harbor seals, respectively. The polymorphisms were largely species‐specific. Although 9,211 of these loci were present in the sequence data of both species (loci determined to be equivalent in the two species if aligned to the same position in the Weddell seal genome), only 560 of the loci were polymorphic in both species.

### 3.1 Historical demography—bottleneck period

With no MAF filter, simulations of past changes in gray and harbor seal population size implemented in DIYABC identified the historical expansion with recent bottleneck scenario (4) as the most likely model in all cases, with a relatively low error rate around 5% (Table 2). In all cases but gray seals at Muskeget Island, the second most likely model was the bottleneck scenario without historical expansion (3), and there was very little to no support found for either of the other scenarios. All summary statistics for the observed datasets fall within the range of the simulated datasets using the defined scenarios and priors, confirming that the selected models do fit the observed data well.

**Table 2:** Likelihood (± 95% confidence interval) for each of four historical demographic scenarios, as depicted in Figure 3, for three gray seal breeding colonies and harbor seals by‐caught in the Northeast United States. For each sample set, demographic models were tested using datasets with and without a minor allele frequency (MAF) filter of 0.05. Error rates calculated as the probability of selecting the given scenario as most likely if it is incorrect

	Scenario 1		Scenario 2		Scenario 3		Scenario 4	
	Likelihood (95% CI)	Error rate	Likelihood (95% CI)	Error rate	Likelihood (95% CI)	Error rate	Likelihood (95% CI)	Error rate
Without MAF								
Gray seal								
Gulf of St Lawrence	0.24 (0–1.07)	3.63	0 (0–0)	0.17	45.23 (44.23–46.23)	3.83	54.53 (53.53–55.53)	5.00
Sable Island	0.05 (0–0.52)	3.87	0 (0–0)	0.10	31.68 (30.69–32.69)	4.37	68.26 (67.26–69.26)	5.50
Muskeget Island	21.16 (20.05–22.26)	3.60	0 (0–0)	0.10	14.44 (13.62–15.26)	4.40	64.4 (63.32–65.59)	5.37
Harbor seal Northeast United States	0 (0–0.77)	3.63	0 (0–0)	0.10	43.97 (42.99–44.96)	3.37	56.02 (55.04–57.00)	4.90
With MAF								
Gray seal								
Gulf of St Lawrence	73.8 (72.93–74.67)	10.30	0 (0–0)	0.73	16.63 (15.90–17.37)	6.83	9.57 (8.99–10.15)	3.10
Sable Island	87.06 (86.40–87.72)	9.67	0 (0–0)	0.97	9.04 (8.47–9.60)	7.13	3.9 (3.52–4.28)	3.70
Muskeget Island	82.37 (81.62–83.12)	8.73	0 (0–0)	0.63	9.36 (8.79–9.94)	7.57	8.26 (7.72–8.81)	3.50
Harbor seal Northeast United States	0.07 (0–0.46)	8.03	0 (0–0)	0.70	27.02 (25.96–28.08)	7.27	72.91 (71.85–73.97)	4.43

When a minimum MAF filter of 0.05 was implemented, the bottleneck scenarios remained most likely only for harbor seals from the Northeast United States (Table 2). The null hypothesis of no change in population size (model 1) could not be rejected for any of the gray seal samples. Error rates were generally higher for all models under the MAF filter condition.

**Figure 5:**
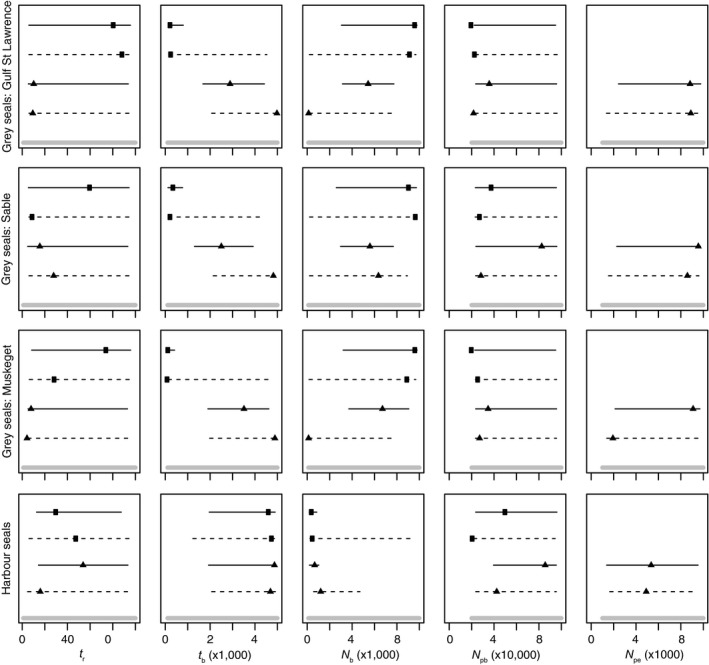
Mean (± 95% confidence interval) posterior parameter estimates for scenarios 3 (square) and 4 (triangles) evaluated using 1000 SNPs with (dashed) and without (solid) a minimum minor allele frequency filter of 0.05. Gray lines at the bottom of each plot represent the prior. Scenarios and parameters as depicted in Figure 3. *t*_*r*_: time of recovery, *t*_*b*_: time of bottleneck, *N*_*b*_: population size during bottleneck; *N*_pb_: population size prior to the bottleneck; *N*_pe_: population size prior to the expansion

Our data had limited power to precisely estimate model parameters related to the timing of population bottleneck or expansion, or the effective population sizes at various points in history. With few exceptions, the 95% confidence intervals around most posterior estimates were broad, encompassing much of the prior range, and therefore deemed uninformative (Figure 5). The exceptions included gray seal bottleneck timing (*t*_*b*_) and the magnitude of the harbor seal bottleneck (*N*_*b*_). In these cases, the data suggest the gray seal bottleneck occurred very recently, and the harbor seal population was reduced during the bottleneck by ~2.5 orders of magnitude from the current population size.

### 3.2 Historical demography—recovery period

Throughout and following recovery, we find little to no significant increase in genomewide diversity among gray seal cohorts sampled from 1973 to 2015 on Sable Island (A_R_: *F*_4_ = 0.590, *p* = .670; H_E_: *F*_4_ = 0.617, *p* = .650) and from 2002 to 2016 on Muskeget Island (A_R_: *t* = −0.533, *p* = .594; H_E_: *t* = −3.823, *p* < .001), or among harbor seal cohorts sampled from 1991 to 2015 (A_R_: *F*_2_ = 5.936, *p* < .01; H_E_: *F*_2_ = 0.2, *p* = .819) (Table 1). We do, however, observe significant deviations in the rate of allele frequency change over this time period of recovery in comparison with neutral expectations under drift during logistic population growth for Sable Island gray seals and Northeast United States harbor seals. In comparison with simulations parameterized with species‐specific estimated population sizes and growth rates from the literature, and observed initial allele frequencies from the oldest cohort, we observe 96 gray seal loci and 98 harbor seal loci with significant (*p* < .05) trends in allele frequency change over time that exceed the maximum rate of change in all simulated loci. The overall variance in rates of change in allele frequency among cohorts in both cases was significantly greater for observed loci than simulated loci (gray: *F* = 11, *p* < 1 × 10^15^; harbor: *F* = 478, *p* < 1 × 10^15^) despite no significant difference in the average rate of change in allele frequency (mean slope = 0.00; gray: *t* = 0.48, *p* = .6; harbor: *t* = −0.76, *p* = .4).

We found no significant difference in measures of genomic diversity among gray seal colonies when cohorts were combined (A_R_: *F*_2_ = 2.04, *p* = .13; H_E_: *F*_2_ = 0.025, *p* = .975) (Table 1). There was no evidence to support significant genetic population structure following spatial expansion for either species (*K* = 1 for the most recent cohorts), and a hierarchical AMOVA identified little variation that could be explained at the level of cohort (0.15%, *F*_SC_ = 0.00149, *p* < .05) or colony (0.03%, *F*_CT_ = 0.00027, *p* = .10) for gray seals, where both levels were sampled.

## 4 DISCUSSION

One advantage of long‐term studies is the ability to track ongoing evolutionary processes in natural populations, which harbor biological variance that cannot be replicated in laboratory settings. Here, we assess the utility of a genotyping‐by‐sequencing approach for cross‐species analyses of such evolutionary processes in two nonmodel species that lack reference genomes, but share demographic histories. With ddRAD sequencing, we investigate sympatric populations of gray and harbor seals in the Northwest Atlantic that provide parallel natural experiments of historical decline and subsequent recovery. Through this analysis, we add to a growing body of literature on the importance of exploring parameter space in genotyping‐by‐sequencing and demographic analyses. Many of the studies in this area thus far have drawn conclusions from evaluations of simulated data. Here, we highlight the value of evaluating such analytical approaches with empirical data as well, using cases of well‐described demographic history that accurately represent the variation inherent in data from samples of natural populations.

Our study contributes to a small, but rapidly growing number of RAD studies that compare data across, rather than or in addition to within, species and genera. These studies, using both simulated and empirical data, have quantified the number of retained homologous loci among species with varying divergence dates. Simulations suggest that hundreds of orthologous, phylogenetically informative loci can be identified in species with divergence times of up to 60 million years (Cariou, Duret, & Charlat, 2016a,2016b; Rubin, Ree, & Moreau, 2012), but empirical studies have successfully applied RADseq to phylogenetic studies of species only from more recent radiations (<100,000 years; Keller et al., 2013; Wagner et al., 2013) and find that the number of shared SNPs drops, sometimes quickly, as divergence dates increase (Lexer et al., 2013; Pante et al., 2015). Among our two studied seal species, harbor and gray, that diverged approximately 5 mya (Arnason et al., 2006), we found over 100,000 homologous RAD loci. This level of observed overlap is comparable and intermediate to that which has been previously described for other interspecific comparisons (Lexer et al., 2013; Pante et al., 2015; Viricel, Pante, Dabin, & Simon‐Bouet, 2014). For example, in one other RADseq study of two marine mammals, Viricel et al. (2014) found that 66% of their identified loci were shared between the short‐beaked common dolphin (*Delphinus delphis*) and the harbor porpoise (*Phocoena phocoena*) that diverged 14–19 mya. The variation in how quickly the number of homologous RAD loci declines with increasing divergence between species could be due to differences in sequencing protocols or analytical pipelines (though Pante et al. (2015) reports little change in the number of homologous loci across multiple pipelines), but more likely reflects true differences in rates of molecular evolution across lineages. Our pinniped study and Viricel et al.'s (2014) cetacean study are more promising in the applicability of RADseq to studies of divergent species, while Pante et al. (2015) findings of lower than 30% shared loci in octocoral species that diverged only 1–2 mya offer a cautionary note. Ultimately, pilot testing of RADseq protocols across multiple study species is necessary to evaluate their potential for generation of large‐scale SNP datasets for interspecific analyses.

Among the relatively large number of shared homologous loci between gray and harbor seals, we found clear differences in the rates of polymorphism. While optimizing the parameters for ddRAD sequence analysis with a representative subset of individuals of equal size from both gray and harbor seals, it became evident that the contemporary genomes of gray seals were generally more diverse than those of harbor seals in the Northwest Atlantic. While both datasets included similar numbers of loci and sequences per individual, many more polymorphic loci and SNPs were identified among gray seal loci than harbor seal loci (Figure 4). We acknowledge that there exist several sources of bias in estimates of genetic diversity from RAD sequencing data (Arnold, Corbett‐Detig, Hartl, & Bomblies, 2013; Cariou, Duret, & Charlat, 2016a,2016b; DaCosta & Sorenson, 2014; Davey et al., 2013; Gautier et al., 2013), but contend that relative comparisons of diversity between species for which data have been collected and processed in a similar manner should be robust (Lozier, 2014).

The observed difference in genomic diversity between gray and harbor seals may be attributable to differences in population size today or historically. Today, the gray seal population in the Northwest Atlantic is significantly larger (>500,000) than the harbor seal population (~85,000) (COSEWIC, 2007; Waring et al., 2016). A discussion of our investigation of historical changes in population follows in the subsequent section. The differences may also be due to the wider geographic range sampled for gray seals in comparison with harbor seals, but our findings of no significant structure or differences in diversity between gray seal colonies do not strongly support this potential explanation. Although differences in species diversity have not been previously observed in microsatellite markers assessed in both species (Goodman, 1998; Worthington Wilmer et al., 1999), one study of genetic diversity at the immune gene complex, MHC class I, in gray and harbor seals reports preliminary findings of greater diversity in gray seals in a small number of individuals (Hammond, Guethlein, Norman, & Parham, 2012). If harbor seals are truly less genetically diverse than gray seals at immune markers and/or more broadly across the genome, this may, in part, explain general observations of lower disease resistance among harbor seals than gray seals (Bogomolni, 2014), an avenue of research that requires further investigation of diversity in functional regions of the genome at a population or species‐wide scale.

### 4.1 Historical demography—bottleneck period

Despite these differences in variability of the two species, we find that signatures of a historical population bottleneck remain evident in the contemporary genomes of both gray and harbor seals following their recovery (Table 2). Varying rates of immigration, emigration, and recent population growth rates among the three gray seal breeding colonies do not appear to affect the power of this approach to detect historical bottlenecks. Beyond the recent bottleneck occurring during the time of human contact, we also find evidence of a historical expansion following the end of the last glacial maximum. Across four demographic models representing all possible combinations of presence and absence of the expansion and bottleneck events (Figure 3), our findings suggest the bottleneck event is the strongest signal in the data. The model identified as most likely in all cases incorporated both the bottleneck and expansion (4), and we found moderate support for the bottleneck only model (3) and little to no support for the expansion only model (2). In fact, we find more than a 75% (and more than 99% in all but one case) likelihood for bottleneck occurrence, when the two models with bottleneck components are considered together, and a lower than 5% probability that these models were incorrectly identified as most likely.

The above discussion refers to analyses carried out without a MAF filter. Similar to prior ABC sensitivity analyses to bioinformatic processing (Shafer et al., 2015), we find that MAF filters increase the error rates associated with model selection and reduce the power of the analyses to reject the null hypothesis scenario of no change in population size. Given the expectation of an excess of rare genetic variants in populations that have experienced recent rapid growth (Keinan & Clark, 2012; Maruyama & Fuerst, 1985a,1985b), it is perhaps unsurprising that parameters which increase our ability to detect rare genetic variants perform better for our objectives. However, this point is important to highlight given the continued frequency of MAF filters in genotyping‐by‐sequencing studies.

Our finding of a demographic bottleneck in gray seals differs from that of Klimova et al. (2014) who reported greatest support for models of postglacial expansion, rather than recent bottleneck, for gray seals across the North Atlantic. This comparison may be a case‐in‐point of the possible variation in outcomes with different sample design and/or analytical pipelines. Klimova et al. (2014) analyzed 350 bp of the mitochondrial hypervariable region and nine microsatellites in gray seals from Sable Island. Prior evaluations of DIYABC with simulated datasets have suggested that SNP data have greater power to recover correct scenarios than mitochondrial or microsatellite data, alone or combined (Cabrera & Palsbøll, 2017). Yet, few empirical studies have used genotyping‐by‐sequencing data (e.g., RADseq, GBS) to evaluate signatures of bottlenecks, and fewer yet have compared the effectiveness of bottleneck detection using RAD sequencing and other markers. Those that do also report variable conclusions across marker types. In declining and stable populations of bumble bees (*Bombus impatiens* and *B. pensylvanicus*), Lozier and colleagues detected population bottlenecks using both microsatellites (Lozier, Strange, Stewart, & Cameron, 2011) and RADseq (Lozier, 2014), but report a difference in relative microsatellite and RADseq diversity between species. Similar to our findings in the gray seal, Shafer et al. (2015) detected signatures of a genetic bottleneck in GBS data that were absent in microsatellite analyses of the Atlantic walrus (*Odobenus rosmarus rosmarus*) (Andersen et al., 2009). Jeffries et al. (2016) also report low power of microsatellites compared to RAD sequencing data to reconstruct the historical phylogeography of the Crucian carp (*Carassius carassius*). In fact, microsatellite studies often fail to detect bottlenecks in populations with histories of decline, an issue which has been generally attributed to limited statistical power, low sample sizes, and underestimation of microsatellite mutation rates (Peery et al., 2012).

Beyond detection of bottlenecks (i.e., model selection), ABC analyses are attractive in their potential to estimate model parameters such as historical population sizes and timing of demographic events. Yet, similar to prior evaluations of ABC approaches using simulated data (Cabrera & Palsbøll, 2017; Shafer et al., 2015), we found our empirical data provided poor resolution in posterior parameter estimates. Most of the estimates were uninformative with broad confidence intervals that spanned much of the prior range (Figure 5). For example, there was disappointingly little evidence from which we could estimate prebottleneck population size and thus cannot resolve debates on whether or not current populations are “overabundant” or simply recovering to pre‐exploitation population size. The few exceptions where model parameters can be confidently estimated from our data do appear consistent with expectations and reflect a recent bottleneck in gray seals and a bottleneck of ~2.5 orders of magnitude in harbor seals. Our power to detect a bottleneck of this size is consistent with Cabrera and Palsbøll's (2017) findings of high likelihood of correct bottleneck scenario detection when a simulated bottleneck is two orders of magnitude in size (compared to little to no power to detect bottlenecks of lower magnitude).

### 4.2 Historical demography—recovery period

In addition to the deep historical analysis of the bottleneck period, multidecadal sampling of several sites in our study area enabled an investigation of the return of genomic diversity as populations recover. Although we did not detect any measurable change in genome‐wide diversity among cohorts, we did observe greater variance and degree of allele frequency change over time than predicted by a neutral model of allelic drift under scenarios of population growth. These differences between observed and simulated data may reflect differences between the true and estimated population growth rates and effective population sizes measured in the field, which are used to parameterize the model, or deviations from the assumptions of the model.

For example, the Wright–Fisher model of genetic drift assumes that all individuals contribute equally to subsequent generations and assumes that all loci are neutral (i.e., no influence of selection on which alleles are passed from one generation to the next). Yet, we know from prior studies that gray seals exhibit a polygynous mating system with relatively high reproductive skew (Worthington Wilmer et al., 1999), violating the first assumption. Furthermore, given the assumption of neutrality, it is unsurprising that the model does not have the capacity to accurately estimate outlier loci that change in allele frequency more than expected over the sampled study period. These temporal outliers within Sable Island gray seals or the Northeast United States harbor seal population could be considered analogues to alleles that surf a spatial expansion (Klopfstein, Currat, & Excoffier, 2006), or reflect selection for traits that are advantageous in a rapidly growing population. Without an annotated reference genome for either study species, with limited knowledge of linkage disequilibrium, and with measured variation in primarily noncoding regions, our ability to further evaluate these potential signatures of selection is limited in this study system, but the markers represent interesting targets for future study.

As gray seal populations have recovered in the Northwest Atlantic, several new sites have been recolonized and their geographic distribution has expanded. With samples from Canada to Massachusetts, our dataset spans this range expansion. In contrast to theoretical expectations of a linear decline in diversity along the expansion axis due to founder effects (Austerlitz, Jung‐Muller, Godelle, & Gouyon, 1997; Nei et al., 1975), we did not observe any significant difference in diversity between samples collected from Canadian source populations and those from Muskeget Island at the edge of the range expansion. We further find no evidence for population structure within the Northwest Atlantic gray seal population, consistent with prior studies of microsatellite and mitochondrial markers (Cammen et al., 2017; Wood et al., 2011).

The lack of difference in diversity over space could be explained by high rates of migration, which is consistent with rapid population growth on Muskeget Island and the frequency of observations of marked adults originating from Canadian source colonies, or result from limited power given the small number of generations (<10) that can be feasibly sampled in a long‐lived animal. While frequent, ongoing migration could also explain the lack of structure, prior studies of gray seals in the Northeast Atlantic suggest structure can arise following a recent history of colony loss and recolonization (Cammen et al., 2011; Gaggiotti et al., 2002). In fact, in the Northeast Atlantic, the level of structure even on the order of tens of kilometers is such that the source of migrants can be probabilistically determined (Gaggiotti et al., 2002). In this system, structure is attributed to the behavioral tendency of this species for natal philopatry and site fidelity.

The lack of similar structure in the Northwest Atlantic could be explained by environmental differences or differences in the historical time frame of exploitation, recovery, and expansion between regions. While declines due to historical hunting occurred earlier in time in Northern Europe than the United States and Canada, periods of recovery over the last several decades have largely overlapped (Duck & Thompson, 2007; Gaggiotti et al., 2002; Härkönen et al., 2007), and Klimova et al. (2014) do not report differences in modeled parameters of demographic change between the two regions. Given the very recent nature of recovery and expansion of gray seals, it is possible that newly recolonized areas remain in nonequilibrium conditions and sufficient time has not yet passed to result in appreciable allele frequency divergence via drift in a substructured population. If this is the case, continued monitoring may find genetic structure in the future. However, it is also possible that differences in the availability and geographic distribution of breeding areas may underlie stable differences in the degree of genetic structure between the two regions. In particular, the gray seal metapopulation in the Northeast Atlantic is composed of many, relatively small, well‐established breeding areas (Duck & Thompson, 2007; Gaggiotti et al., 2002; Härkönen et al., 2007), while the vast majority of breeding in the Northwest Atlantic takes place at a single colony on Sable Island, where current annual pup production is estimated to exceed 80,000 (Hammill et al., 2017). If most of the other breeding areas in the Northwest Atlantic (excluding the well‐established sites in the Gulf of St. Lawrence), which are in the process of recolonization, have been largely founded by immigrants from a single central source, we cannot expect to detect genetic structure.

In this system of sympatric pinnipeds in the Northwest Atlantic, which remains in flux and lacks solid baselines, the historical genetic analyses presented here provide one additional line of evidence that can be considered when deciding how to manage recovering protected species. The use of genetics to describe contemporary genetic structure is not novel, but the in‐depth exploration of historical diversity offers further insights. Despite significant differences in the degree of genetic diversity measured using the selected ddRAD technique among gray and harbor seals, signatures of their shared demographic history (bottlenecks) are readily apparent in the contemporary genomes of both species. The models were unfortunately unable to shed much light on prebottleneck population sizes, which is of particular interest in this system that is currently struggling with perceptions of overabundance (Roman, Dunphy‐Daly, Johnston, & Read, 2015), but the genomic data appear otherwise consistent with archeological and historical records. Through comparison to this rich, though at times uncertain, description of the history of these two species, we highlight precision of parameter estimates, such as timing of demographic events and degree of changes in population size, as well as the processes that drive genetic diversity during recovery, as key areas for potential growth in current analytical pipelines.

## CONFLICT OF INTEREST

None declared.

## AUTHOR CONTRIBUTION

K.M.C. designed and performed the research and analyzed the data. T.F.S. contributed methodological assistance, reagents, and analytical tools. W.D.B., M.O.H., W.B.P., J.R., F.W.W., and S.A.W contributed samples. T.R.F. and M.K. supervised the study. K.M.C. wrote the manuscript with input from all authors.

## DATA ACCESSIBILITY

RAD loci sequences are available in the NCBI Short Read Archive (Accession no. SRP140440). Results of the Stacks analysis are archived at Dryad https://doi.org/10.5061/dryad.fg52075.
