# Supplementary material for: Genomic signatures of population bottleneck and recovery in Northwest Atlantic pinnipeds
Source: Ecol Evol. 2018 Jun 5;8(13):6599–614. doi: 10.1002/ece3.4143 (PMC6053562; doi:10.1002/ece3.4143)
Supplement: Supplementary file 1 [file ECE3-8-6599-s001.pdf]

Supporting Information

Genomic signatures of population bottleneck and recovery in Northwest Atlantic pinnipeds

Kristina M. Cammen[1]*, Thomas F. Schultz[2], W. Don Bowen[3], Michael O. Hammill[4], Wendy B. Puryear[5], Jonathan Runstadler[5], Frederick W. Wenzel[6], Stephanie A. Wood[7], Timothy R. Frasier[8], Michael Kinnison[9]

[1]School of Marine Sciences, University of Maine, Orono, ME 04469, USA; [2]Duke University Marine Lab, Beaufort, NC 28516, USA; [3]Bedford Institute of Oceanography, Dartmouth, NS B2Y 4A2, Canada; [4]Fisheries and Oceans Canada, Maurice Lamontagne Institute, Mont-Joli, QC G5H 3Z4, Canada; [5]Cummings School of Veterinary Medicine, Tufts University, North Grafton, MA 01536, USA; [6]Protected Species Branch, NOAA, NMFS, Northeast Fisheries Science Center, Woods Hole, MA 02543 USA; [7]Department of Biology, University of Massachusetts, Boston, MA 02125 USA; [8]Department of Biology, Saint Mary's University, Halifax, NS B3H 3C3, Canada; [9]School of Biology and Ecology, University of Maine, Orono, ME 04469, USA

*Corresponding author: kristina.cammen@maine.edu

Supplemental Methods

We conducted double-digest RADseq following the protocol described in Peterson et al. (Peterson *et al.* 2012) with minor modifications. Briefly, 100ng of genomic DNA was double-digested with 10U each of SBfI-HF® and MspI restriction enzymes (New England Biolabs) at 37 °C for 2 h. One of 32 individually 6-bp barcoded P1 adaptors and a single P2 adaptor were ligated to the SbfI and MspI sites, respectively, to facilitate multiplexing of 32 individuals per library. Ligation reactions were carried out in 40ul reaction volumes with 6.25nM each of P1 and P2 adaptors, 1.25mM rATP, 1x reaction buffer, and 200U concentrated T4 DNA ligase (New England Biolabs), incubated at room temperature for 2 h. Thirty-two samples with unique barcodes could subsequently be pooled prior to library amplification, which was carried out in a 50ul total reaction volume containing 5ul library DNA, 0.5uM each of P1 and P2 primers, and 1x Phusion® High-Fidelity PCR Master Mix (New England Biolabs). One of two P2 primers with unique 6-bp indexes were used to facilitate multiplexing of two libraries per sequencing lane, for a total of 64 individuals per lane. Amplification was conducted using the following temperature cycle: 30 s 94 °C, 30 s 58 °C, 1 min 72 °C, 18 cycles. DNA purification using magnetic 1.5x beads (Sera-Mag SpeedBeads) was carried out at multiple time points throughout this process following digestion, ligation, and amplification.

Peterson BK, Weber JN, Kay EH, Fisher HS, Hoekstra HE (2012) Double digest RADseq: an inexpensive method for *de novo* SNP discovery and genotyping in model and non-model species. *PloS One* **7**, e37135.
